# Trauma Mechanisms and Injuries Associated with Go-Karting

**DOI:** 10.2174/1874325001004020107

**Published:** 2010-02-17

**Authors:** Hasan H. Eker, Esther M.M. Van Lieshout, Dennis Den Hartog, Inger B. Schipper

**Affiliations:** Department of Surgery-Traumatology, Erasmus MC, University Medical Center Rotterdam, Rotterdam, The Netherlands

**Keywords:** Trauma, go-kartig, spine.

## Abstract

Annually, approximately 600 patients seek medical attention after go-kart accidents in the Netherlands. A large variability in injury patterns can be encountered. Knowledge of the trauma mechanisms of go-kart accidents and insight into the associated injuries is limited and requires improvement. Such additional knowledge may lead to customized trauma protocols for patients with a high index of suspicion on go-kart injuries. Research into trauma mechanisms may also lead to implementation of improved or additional safety measures for go-karting, involving both the go-karts itself as well as prerequisites to the go-kart tracks and qualifications for the drivers. The main trauma mechanisms involved in go-kart accidents, and three cases to illustrate the variety of injuries are described in the current manuscript.

## INTRODUCTION

Since its introduction in the 50's, go-karting has strongly developed itself as sports and leisure opportunity. Indoor and outdoor go-karting tracks accommodate thousands of people annually. Some even consider go-karting a stepping stone towards professional Formula 1 sports [1].

**Fig. (1):**
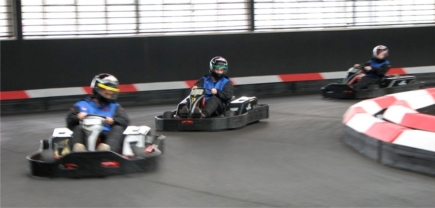
**Karting at an indoor track.** Trunk and extremities are relatively unprotected.

While sitting in a go-kart, the trunk and extremities are relatively unprotected (Fig. **1**). The accident rate during go-karting is exceedingly high. Despite the substantial risk of severe injuries, the number of mandatory safety requirements for this sport remains low. As opposed to other sports that involve motorized vehicles such as motocross or Formula 1, implementations of safety measures by the manufacturers and managers of go-karting tracks are not mandatory in most cases. Compared with other high-risk sports hardly any scientific data is available about injuries and risks concerning go-karting; this might explain the limited number of safety requirements.

In medical literature mainly case reports, without explicitly describing the underlying trauma mechanism are presented. However, Govaert *et al*. published a case report on fractures of the pancreas after a go-kart accident in 2001 [2]. In this study the trauma mechanism was a blunt-force abdominal trauma of the steering wheel of the go-kart. An expert panel of three (trauma) surgeons has been established aiming to analyze the risks associated with go-karting. Following critical analysis of the sports itself as well as the materials used, the expert panel identified two main groups of factors that determine the trauma mechanism: Factors related to the materials used and velocity-related factors. Based upon this the following three categories of trauma mechanisms have been defined (Table **1**).

1. Injuries to the extremities and trunk that are related to a direct collision at either side of the go-kart; these injuries are mainly associated with cuts, bruises, and fractures.
2. High-energetic trauma, mainly caused by frontal collision; this usually causes blunt-injury abdominal or thoracic injuries, but also compression fractures of the lower extremities can be seen.
3. Acceleration/deceleration trauma causing hyperextension injuries to the (cervical) spine.

These trauma mechanisms may result in severe injures. In the Netherlands, approximately 600 persons are being treated at the Emergency Department after a go-kart accident each year [3]. This number is most likely an underestimation of the actual number of patients, since patients who did not seek treatment at an Emergency Department of a hospital were not included in these statistics. The injuries resulting from go-kart accidents are very heterogeneous [4,5,6]. A mean length of hospital stay of 32.5 days illustrates the complexity and severity of the associated injuries [7].

In the years 2000 to 2006 twelve patients with severe injuries after a go-kart accident have been treated at the Erasmus MC. Three cases illustrating the variability in trauma mechanisms and injuries are discussed below.

## PATIENT A

A 17-year-old woman reported to the Emergency Department with upper abdominal pain. Earlier that day she had been involved in a high-speed frontal collision to the guard-rail during go-karting, causing blunt-force trauma to her abdomen by the steering wheel.

In the emergency room the patient complained about abdominal pain and no other abnormalities were found elsewhere. Serum amylase levels were slightly elevated. An initially performed ultrasound scan of the abdomen showed a contusion of the pancreas. During the course of the day, her abdomen became increasingly painful. At physical examination a diffuse painful abdomen was observed; no abnormalities were found elsewhere. Serum amylase levels were slightly elevated. Computed tomography (CT), indicated in order to exclude additional injuries, subsequently showed a complete rupture of the pancreas at the transition of the pancreatic head to the corpus.

**Fig. (2):**
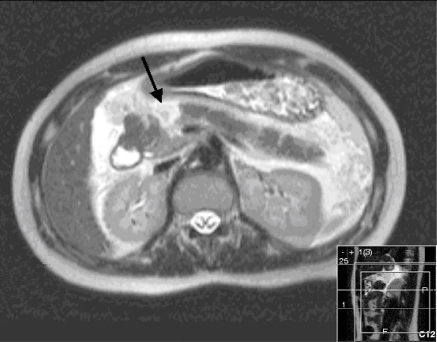
**Magnetic Resonance CholangioPancreatography of patient A.** The black arrow indicates the pancreatic fracture.

During observation in the surgical ward a complementary MRCP (Magnetic Resonance Cholangio Pancreatography) was performed which confirmed the CT-diagnosis and was the indication for operation (Fig. **2**). At laparotomy a fracture of the pancreas was seen at level of the corpus of the pancreas. The pancreatic head was closed with a running suture and a pancreaticojejunostomy was performed to the distal part of the pancreas using a Roux-en-Y jejunal loop. A superficial wound infection complicated the postoperative recovery. After treatment of the wound infection she could be discharged.

## PATIENT B

A 50-year-old man was directed by a regional hospital to our Emergency Department. The patient sustained a high-speed frontal collision with his go-kart to the guard-rail earlier that day. After the accident he was unable to weight-bear his foot and complained about severe pain. The initial X-ray revealed a fracture of the calcaneus. The diagnosis was confirmed by a 3D CT-scan showing a multifragmentary dislocated intra-articular calcaneal fracture. Closed reduction and percutaneous fixation was performed using three cannulated screws. The patient was discharged without any postoperative complications.

## PATIENT C

**Fig. (3):**
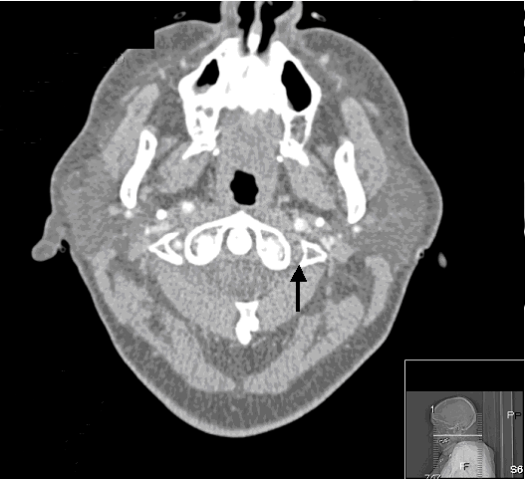
**Computed Tomography Angiogram of patient C.** The black arrow indicates a dissection of the left vertrebrate aretery as a result of deceleration trauma.

A 49-year-old woman was admitted to the Emergency Department of our hospital because of persisting pain in her neck after a go-kart side impact accident. She also complained about asymmetry of her facial muscles, difficulty in articulating words, pain in her left shoulder and right ankle. Initially a Computed Tomography Angiogram was performed, showing a dissection of the left vertebral artery (Fig. **3**). This dissection was treated conservatively with a platelet aggregation inhibitor (carbasalate calcium 100 mg). On additional X-Rays a fracture of the left clavicle, a fracture of the medial sight of the right talus, and an impression fracture of the navicular bone were diagnosed. The fracture of the talus was operated by open reduction and internal fixation with screws of the talus. The clavicular fracture was treated with a sling for three weeks and painkillers. No complications occurred during his admission or rehabilitation.

## DISCUSSION

Injuries resulting from go-kart accidents can vary widely in severity, resulting in great differences in outcome. An overview of annual numbers of admissions to Dutch Emergency Department and injuries after go-kart accidents divided into different anatomic regions is shown in (Table **2**). Superficial injuries and bruises of the trunk and limbs as an effect of direct trauma are commonly encountered. Fractures are found less frequently, but are diagnosed in 24% of all cases presenting to the Emergency Department [3,4]. Interestingly, a substantial part of the patients (39%) have injuries to their trunk or spine as an effect of blunt direct trauma or hyperflexion/extension. These numbers are comparable with the numbers of traffic accidents (30-65%) [8,9].

Some sports are associated with specific types of injuries, e.g. fractures of the distal radius are frequently seen in inline skating. Such an association could not be found for go-kart accidents [10]. This is most likely because of the great diversity of trauma mechanisms with go-kart accidents.

The relatively high injury rate after go-kart accidents could be due to several factors. The driver protection to external forces on the flanks but also at the front is very limited, and go-karts are not always equipped with a standard safety belt. Also, the maximum motor power en speed of indoor go-karts is not subjected to regulations or requirements and both the go-karts as well as the tracks are preferably composed of sustainable materials. A great part of these hard and stiff materials are not very driver friendly in accidents. Another factor that may dictate the accident rate is the fact that go-kart drivers are frequently relatively young and have little experience with driving a vehicle. No driver’s license or certificate is required to operate a go-kart, and for some drivers it is even their first driving experience. These presumptions may explain the fact that almost half of the victims are younger than 24 years [3].

A widely applied safety measure is the safety helmet. The relatively low incidence of head and neck injuries, compared with traffic accidents, may be attributed to the mandatory us of a safety helmet. The introduction of the safety helmet for motorcycle drivers in the past has also shown a significant reduction in head and neck injuries [11].

Despite the relatively high numbers of injuries related to go-kart accidents, only one manuscript linking trauma mechanism of go-karting accidents to injury patterns has been published [2]. By better understanding the trauma mechanisms and types of injuries more passive and active safety measures can be implemented in both the go-karts as well as the go-kart tracks. The three-point safety belt serves as a good example in this respect; it would block hyperflexion of the trunk and may prevent blunt abdominal trauma induced by collapse on the steering wheel. Moreover, specific trauma mechanisms should raise a “high index of suspicion” for specific injuries, which may help to optimize treatment strategies at the Emergency Department. An overview as given in Table **1** could be used to support such thinking. An extensive registration and more research are needed to further study the relationship between trauma mechanisms and specific injuries.

## Figures and Tables

**Table 1:** Three Main Groups of Trauma Mechanisms and Related Injuries

Trauma Mechanism	Injury Type	Specific Injury
Direct trauma	Fracture, contusion, abrasion, laceration, burn wound	Calcaneal or ankle fracture caused by pedals; humeral, shoulder, or clavicular fracture after side impact; wrist or hand fracture by steering wheel
HET deceleration trauma	Blunt abdominal or thoracic trauma, compression injury to the lower extremity	Rib fracture, lung contusion, pneumothorax, fladder thorax, cor contusion, diaphragm rupture; rupture or contusion of spleen, liver, gal bladder, pancreas, kidney, or intestines; fracture or luxation of foot, lower limb, hip, or pelvis
Acceleration/deceleration	Flexion/extension injury	Compression fracture of the spine, whiplash, injury of the carotid or vertrebral arteries

HET, High Energetic Trauma.

**Table 2:** Annual Number of Emergency Department Admissions and Injuries after Go-Kart Accidents Divided into Different Anatomic Regions

	Number	%

Head/neck	70	12
Superficial injury/bruise head	30	5

Trunk/spinal column	230	39
Superficial injury/bruise trunk	160	28
Fracture thorax/rib	30	6

Shoulder/arm/hand	130	22
Superficial injury/bruise shoulder/arm	30	5
Fracture hand/finger	30	5

Hip/leg/foot	150	26
Superficial injury/bruise hip/leg	40	7

Other	20	3

Total	600	100

Source: Letsel Informatie Systeem 2001-2005, Consumer Safety Institute (Amsterdam, the Netherlands) [3].
